# The evaluation of effectiveness of IL-17 and IL-23 inhibitors on nail and enthesis involvement in early psoriatic arthritis patients by high-frequency ultrasonography: a single-centre prospective proof-of-concept study

**DOI:** 10.1007/s11547-025-02086-9

**Published:** 2025-09-18

**Authors:** Piero Ruscitti, Camilla Gianneramo, Pierpaolo Palumbo, Manfredo Bruni, Martina Gentile, Sabrina Lanzi, Emanuele Vagnozzi, Alessia Loda, Lina Maria Magnanimi, Maria Concetta Fargnoli, Antonio Barile, Paola Cipriani, Maria Esposito

**Affiliations:** 1 Department of Biotechnological and Applied Clinical Sciences, University of L’Aquila, Delta 6 Building, Via Dell’ Ospedale, PO Box 67100, L’Aquila, Italy; 2 Department of Emergency and Interventional Radiology, San Salvatore Hospital, Abruzzo Health Unit 1, L’Aquila, Italy; 3 Department of Diagnostic Imaging, Area of Cardiovascular and Interventional Imaging, San Salvatore Hospital, Abruzzo Health Unit 1, L’Aquila, Italy; 4 San Gallicano Dermatological Institute, IRCCS, Rome, Italy

**Keywords:** Psoriasis, Psoriatic arthritis, Therapy, Ultrasonography with high-frequency probes

## Abstract

**Purpose:**

To evaluate the effectiveness of IL-17 and IL-23 inhibitors in psoriatic nail and enthesis involvement by ultrasonography with the use of high-frequency probes (HFUS). To correlate the obtained HFUS findings with disease activity of patients with psoriatic arthritis (PsA).

**Material and Methods:**

Consecutive early naïve patients with PsA underwent HFUS on nails and entheses before and after 24 weeks of treatment with IL-17 or IL-23 inhibitor. The Brown University Nail Enthesis Scale (BUNES), considering morphometry and Power Doppler (PD), and the Madrid Sonography Enthesitis Index (MASEI) score were used to evaluate these features. HFUS findings were correlated with the extension of the disease on skin by Psoriasis Area and Severity Index (PASI) and joints by Disease Activity Index for Psoriatic Arthritis (DAPSA).

**Results:**

Twenty early naïve patients with PsA were treated for 24 weeks with an IL-17 or IL-23 inhibitor. A significant reduction of BUNES PD was observed considering the whole cohort of patients receiving these drugs (*p* = 0.044), whereas, despite a trend, no significant difference was reported comparing BUNES morphometry. The BUNES PD correlated with PASI (*r* = 0.466, *p* = 0.030) and with DAPSA (*r* = 0.444, *p* = 0.032), whereas BUNES morphometry did not. A significant reduction of MASEI was observed considering the whole assessed cohort of patients treated with these drugs (*p* = 0.045). The MASEI correlated with both PASI (*r* = 0.429, *p* = 0.037) and DAPSA (*r* = 0.499, *p* = 0.017).

**Conclusions:**

This proof-of-concept study demonstrated that the assessment by HFUS may provide additional accurate information about the effectiveness of IL-17 and IL-23 inhibitors in psoriatic nail and enthesis involvement.

## Introduction

Psoriatic arthritis (PsA) is a chronic inflammatory disease characterized by musculoskeletal manifestations involving peripheral joints, entheses, and axial skeleton in patients with psoriasis (PsO) [1]. PsA is characterized by a heterogeneous clinical manifestations and different disease courses associated with relevant morbidity, disability, and increased rate of comorbidities [2–4]. In a large percentage of patients with PsA, a nail involvement is characterized by a wide spectrum of clinical nail matrix or nail bed pathologic features [5]. However, the psoriatic nail involvement is often underrecognized in the rheumatologic settings even if this is an important predictor of enthesitis and of development of PsA [6]. Considering the pathogenesis of PsA, although not fully elucidated yet, a complex interplay has been proposed including genetic background, triggering environmental factors, and a deregulated immune response [7]. In this context, multiple lines of evidence have recently established the pivotal role of the axis interleukin (IL)-17/IL-23 in PsA, which are pro-inflammatory cytokines acting within a more complex pathogenic network and resulting in the production of further inflammatory mediators and activation of immune cells, fibroblasts, epithelial cells, and synoviocytes [7, 8]. Therefore, drugs inhibiting IL-17 and IL-23 have been successfully used in the treatment of PsA showing clinical benefits on different manifestations of the disease [9, 10]. On these bases, the latest international recommendations for treatment of PsA have recently suggested the administration of biological disease modifying anti-rheumatic drugs (bDMARDs), including IL-17 or IL-23 inhibitors in patients, who are identified as non-responders to the first therapies [11, 12]. In this context, a growing body of evidence suggests the usefulness of ultrasonography in monitoring the response to treatment in PsA [13, 14]. In fact, the ultrasound in B-mode combined with power Doppler (PD) allows the visualization of both morphological and functional changes of involved joints [13, 14]. In addition, the ultrasonography with the use of high-frequency probes (HFUS) may be suggested in accurately determining the clinical response of the involved sites in the area under ultrasound examination [15]. The use of a higher-frequency probe allows a better image resolution and a finer evaluation of assessed structures. Transducers with a frequency higher than 20 MHz are referred to as high-frequency ones, and the obtained images at this frequency range are characterized by superior axial and lateral resolution [15]. In addition, in patients affected by PsO and PsA, we have already shown the usefulness of HFUS in unmasking subclinical abnormalities of nails and enthesis [16]. However, the clinical usability of HFUS in monitoring the response to administered therapies remains to be fully elucidated yet.

On theses bases, we aimed at evaluating the effectiveness of IL-17 and IL-23 inhibitors in psoriatic nail and enthesis involvement by HFUS in a single-centre prospective proof-of-concept study. In addition, we correlated the obtained HFUS findings with disease activity of involved patients to evaluate possible associations.

## Methods

### Study design and patients

Consecutive patients were assessed if fulfilling widely available classification criteria for PsO and PsA from January 2022 to June 2024 [17, 18]. We included adult patients (age > 18 years) with PsO, naïve to systemic immunomodulating therapies, diagnosticated to have PsA with a duration < 2 years, and eligible to treatment with IL-17 or IL-23 inhibitors according to the clinical judgement. Based on these characteristics, we assessed early naïve patients with PsA in the context of PsO in a prospective proof-of-concept study to provide the basis for further specific designed studies. In our cohort, HFUS was performed on nails and entheses of included patients before and after 24 weeks of therapy (detailed below). Experienced dermatologists and rheumatologists clinically evaluated all the included patients; the Psoriasis Area and Severity Index (PASI) and the Disease Activity Index for Psoriatic Arthritis (DAPSA) were used to measure the disease activity in skin and joints, respectively. Patients were not included if not eligible to the administration of bDMARDs including those with uncontrolled comorbidities. The bDMARDs were administered in monotherapy, after the failure of NSAIDs for the joint involvement. Specifically, the IL-17 inhibitors, secukinumab or ixekizumab, were both administered every 4 weeks after the induction phase. Considering the IL-23 inhibitors, following the induction phase for both, risankizumab was administered every 12 weeks, whereas guselkumab every 8 weeks.

The local Ethics Committee approved this study, which was performed according to the Good Clinical Practice guidelines and the latest Declaration of Helsinki. Moreover, patients signed an informed consent allowing the use of clinical and imaging records for scientific purposes.

### General methods of HFUS

The HFUS was carried out by experienced radiologists using high-frequency probes characterized by 27 MHz, which were available on the ultrasound machines (Esaote my Lab X8). All of these had also a software for the implementation to ameliorate the quality of retrieved images with colour Doppler. The imaging features for Doppler ultrasound examinations were set up to increase the evaluation of the low-velocity, low-volume flows within the nail beds as well as the entheses. The PD findings were standardized by a frequency ranging from 10.0 to 12.5 MHz and pulse repetition frequencies ranging from 0.9 to 1.0 kHz; colour gain was set up avoiding a possible excessive colour noise (colour vs. echo priority ranging from 40 to 60% and colour persistence adjusted to high values).

To assess the reproducibility of our measurements, we performed both intra-observer and inter-observer analyses. The intra-observer variability was evaluated by repeating the measurements by the same reader (expert reader, with experience more than 5 years) after an interval of two weeks. For inter-observer variability, measurements obtained by a second independent reader (non-expert reader, with experience more than 2 years) were compared with the averaged measurements of the first reader, due to the high intra-observer concordance.

### The assessment of psoriatic nails by HFUS

All ten nails of the hands were scanned by HFUS to evaluate the possible abnormalities in included patients before and after the treatment. The longitudinal scan identified the nail plate, which was considered to be composed by two hyperechoic lines representing the dorsal and ventral areas with a virtual anechoic space in the between. The nail bed was represented by a hypoechoic band between the superior hyperechoic nail plate and the inferior hyperechoic distal phalanx. The nail matrix was visualized as an isoechoic region under the proximal nailfold at the proximal portion of the nail bed. The Brown University Nail Enthesis Scale (BUNES) evaluated the different nail structures and PD activity as reported in available literature [19]. The normal morphologic images were indicated with a score of 0 for each evaluated area (nail plate, matrix, and bed). The changes of nail plates (focal hyperechoic areas of the ventral plate/irregular border of ventral and dorsal plate), abnormal or thickened nail beds (2.0–3.0 mm), and/or matrix were scored as 1. In addition, the PD images of nail beds and matrixes were assessed as follows: 0 = no signal, 1 = confluent signal in < 25% of the area, 2 = confluent signal in > 25% and < 50%, 3 = confluent signal > 50% [19]. The values of BUNES were expressed as mean of all assessed nails for both morphometry and PD results.

### The assessment of psoriatic entheses by HFUS

In addition, the enthesis abnormalities were assessed by HFUS in all involved patients. The Madrid Sonography Enthesitis Index (MASEI) score assessed the entheses, analysing bilaterally six sites: proximal plantar fascia, distal Achilles tendon, distal and proximal patellar tendon insertion, distal quadriceps tendon, and distal brachial triceps tendon [20]. The evaluation of the tendon thickness was based on the assessor measurement; each tendon was scanned in both planes, the longitudinal and transverse ones. The enthesis evaluation assessed and scored the following features: calcifications, bursae, erosions, thickness, and structure of tendon (cortical bone profile, intra-tendon and paratendon echogenicity) and PD signal in bursa or enthesis. Each item was scored as 1 point, except for calcification (0 = absent, 1 = small calcification or ossification with an irregularity of enthesis cortical bone profile; 2 = clear presence of enthesophytes; 3 = large calcifications or ossifications) erosion, and PD signal (0 or 3 whether they are present or not) as suggested by available literature [20]. The results of MASEI were expressed al mean of scores in all assessed entheses considering both left and right sides.

### Statistical analysis

Statistics firstly provided a descriptive analysis of the retrieved patient data. A comparison of HFUS parameters was made following the administration of IL-17 or IL-23 inhibitors, before and after 24 weeks. Considering the distribution of obtained results, nonparametric tests were performed. After that, Spearman’s correlations were estimated among HFUS parameters, PASI, and DAPSA. The statistical significance was set to *p* < 0.05, and all p-values were two-sided.

Intra-class correlation coefficients (ICCs) were calculated to evaluate both intra-observer and inter-observer reproducibility. A two-way mixed-effects model was applied, considering absolute agreement. ICC values were interpreted as follows: ICC < 0.5 indicating poor reliability, between 0.5 and 0.75 moderate reliability, between 0.75 and 0.9 good reliability, and values greater than 0.9 indicating excellent reliability. Additionally, an ANOVA test was performed to evaluate the possible systematic differences between observers. Statistical analyses were performed by using SPSS software.

## Results

### Clinical features of assessed patients

In this study, 20 early naïve patients with PsA were included and followed up for 24 weeks (14 males and 6 females, mean age 52.1 ± 12.8 years). Clinical characteristics of assessed patients are reported in Table 1. All patients had a moderate to active PsA and were eligible to be treated with bDMARDs. In these patients, 9 out 20 had a clinically evident psoriatic nail involvement. Nail matrix involvement was observed as follows: 8/9 pitting, 7/9 leukonychia, and 1/9 crumbling, whereas nail bed involvement was described as follows: 7/9 onycholysis, 5/9 hyperkeratosis, and 1/9 salmon or oil-drop patches. Among these, 10 patients received IL-17 inhibitors, secukinumab (*n* = 5) or ixekizumab (*n* = 5), whereas 10 received IL-23 inhibitors, risankizumab (*n* = 6) or guselkumab (*n* = 4). All patients but two achieved a good clinical response assessing the skin and the joints with a significant reduction of PASI [baseline 12.0 (11.5) vs 24 weeks 5.0 (6.0), *p* = 0.021], NAPSI [baseline 12.0 (20.0) vs 24 weeks 4.0 (13), p = 0.043], and DAPSA [baseline 24.0 (20.0) vs 24 weeks 10.0 (11.0), *p* = 0.025], respectively. In our cohort, 7 patients were affected by a comorbidity; specifically, 5/20 patients by high blood pressure, 4/20 by hypothyroidism, 2/20 patients from dyslipidaemia, and 1/20 patient by type 2 diabetes. All these comorbidities were stably treated at the time of the present study. No severe side effects leading to the discontinuation of the drug were reported; only transient minor injection site reactions were observed during the 24 weeks of follow-up.

**Table 1:** Clinical characteristics of assessed patients with PsA

Clinical characteristics	20 patients with PsA
Age, years, mean ± standard deviation	52.1 ± 12.8
Male Sex, n (%)	14.0 (70.0%)
Smoking habit, n (%)	8.0 (40.0%)
Obesity, n (%)	6.0 (30.0%)
Comorbidity, n (%)	7.0 (35.0%)
Plaque type PsO, n (%)	18.0 (90.0%)
Inverse PsO, n (%)	4.0 (20.0%)
PASI, median (IQR)	12.0 (11.5)
NAPSI, median (IQR)	12.0 (20.0)
Tender Joints, median (IQR)	8.0 (11.0)
Swollen Joints, median (IQR)	2.0 (2.0)
DAPSA, median (IQR)	24.0 (20.0)
LEI, median (IQR)	2.0 (3.0)
CRP, mg/dL median (IQR)	1.1 (1.2)

PsA, psoriatic arthritis; PsO, psoriasis; PASI, psoriasis areas severity index; NAPSI, nail psoriasis severity index; DAPSA, disease activity in psoriatic arthritis; LEI, Leeds enthesitis index; CRP, C reactive protein

### Reproducibility analysis

The intra-observer ICC demonstrated excellent reliability, with an ICC value of 0.956 (95% confidence interval [CI], 0.899–0.983; *p* < 0.001). The inter-observer ICC similarly indicated excellent agreement, with an ICC value of 0.958 (95% CI 0.896–0.983; *p* < 0.001). However, the ANOVA test for inter-observer analysis revealed a statistically significant difference between observers (*p* = 0.034), suggesting a slight systematic variation.

### The effectiveness of bDMARDS in psoriatic nail involvement by HFUS

**Fig. 1:**
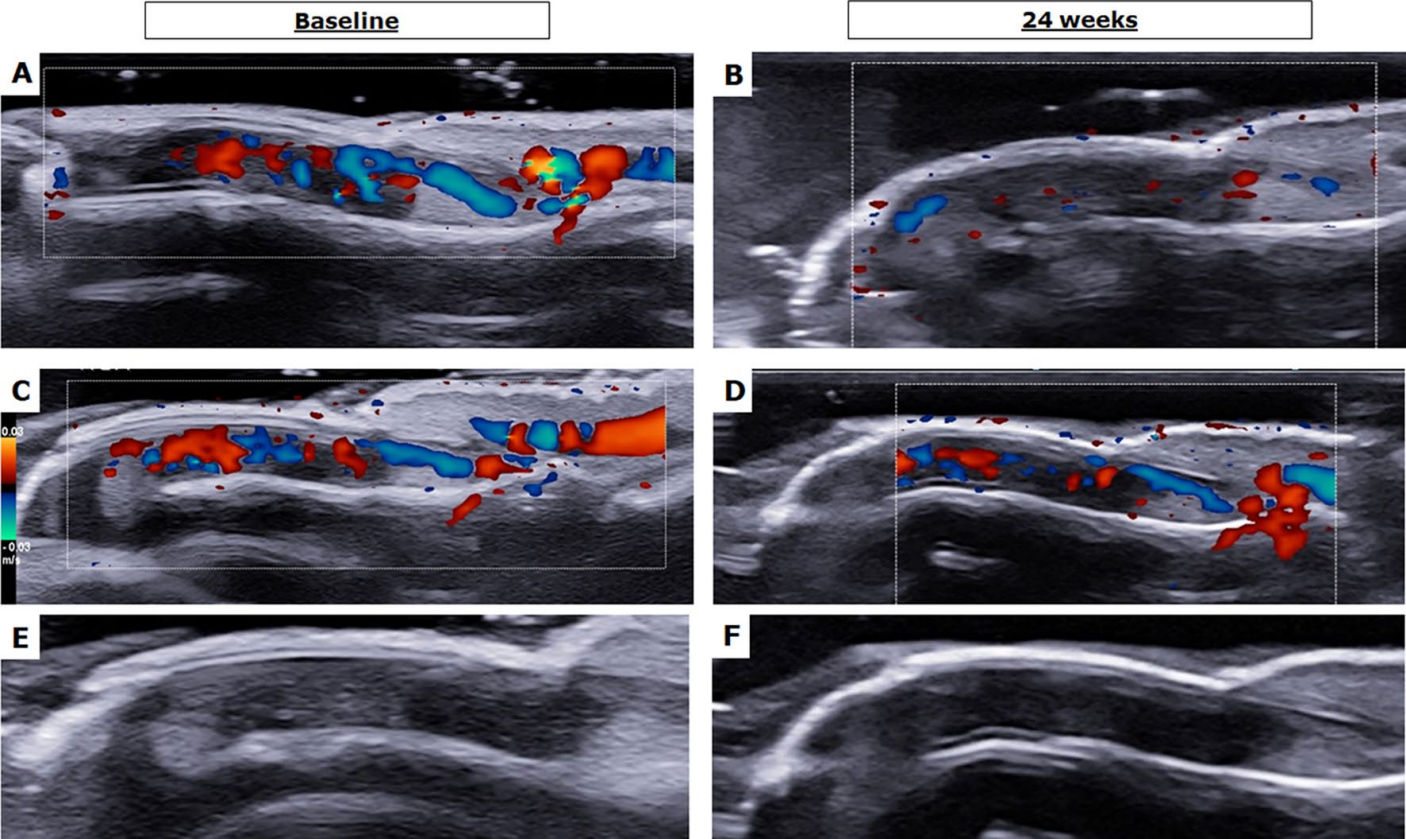
Representative images of nails assessed by HFUS before and after 24 weeks of treatment. In this figure, a relevant reduction of PD in nail matrix and bed may be observed after the treatment in representative evaluated patients as shown in panels **A** and **B** (patient treated with IL-23 inhibitor) and in panels **C** and **D** (patient treated with IL-17 inhibitor). A stabilization of structural changes is reported after the treatment in a representative patient about morphometry of matrix, nail plate, and nail bed as reported in panels **E** and **F** (patient treated with IL-23 inhibitor)

**Fig. 2:**
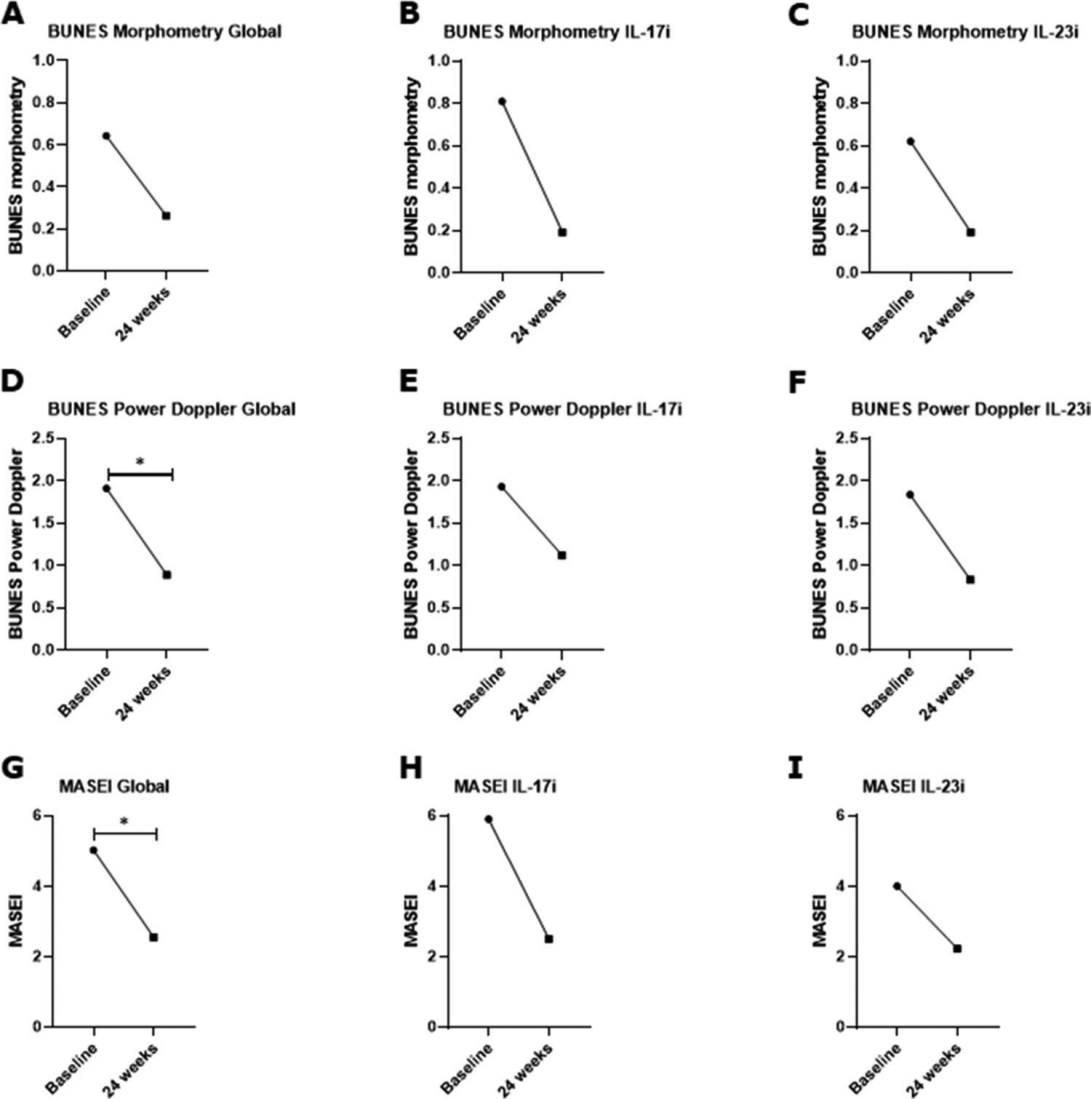
Effectiveness of bDMARDs in psoriatic nail and enthesis involvement evaluated by HFUS by the values of BUNES and MASEI before and after 24 weeks of treatment. In this figure, the effectiveness of bDMARDs in psoriatic nail and enthesis involvement evaluated by HFUS is reported. Despite the trend, no significant reduction of the values of BUNES morphometry was observed before and after the treatment considering the whole cohort of treated patients with bDMARDs (*p* = 0.578), the patients treated with IL-17 inhibitors (*p* = 0.405), and the patients treated with IL-23 inhibitors (*p* = 0.424), as shown in panels **A**, **B**, and **C**, respectively. A significant reduction of the value of BUNES PD was reported before and after the treatment considering the whole cohort of treated patients with bDMARDs (*p* = 0.044), but not assessing the patients treated with IL-17 inhibitors (*p* = 0.315), and the patients treated with IL-23 inhibitors (*p* = 0.119), as shown in panels **D**, **E**, and **F**, respectively. A significant reduction of the value of MASEI was pointed out before and after the treatment considering the whole cohort of treated patients with bDMARDs (*p* = 0.045), but not analysing the patients treated with IL-17 inhibitors (*p* = 0.083), and the patients treated with IL-23 inhibitors (*p* = 0.173), as shown in panels **D**, **E**, and **F**, respectively. All these results are expressed as median and range inter-quartile

**Fig. 3:**
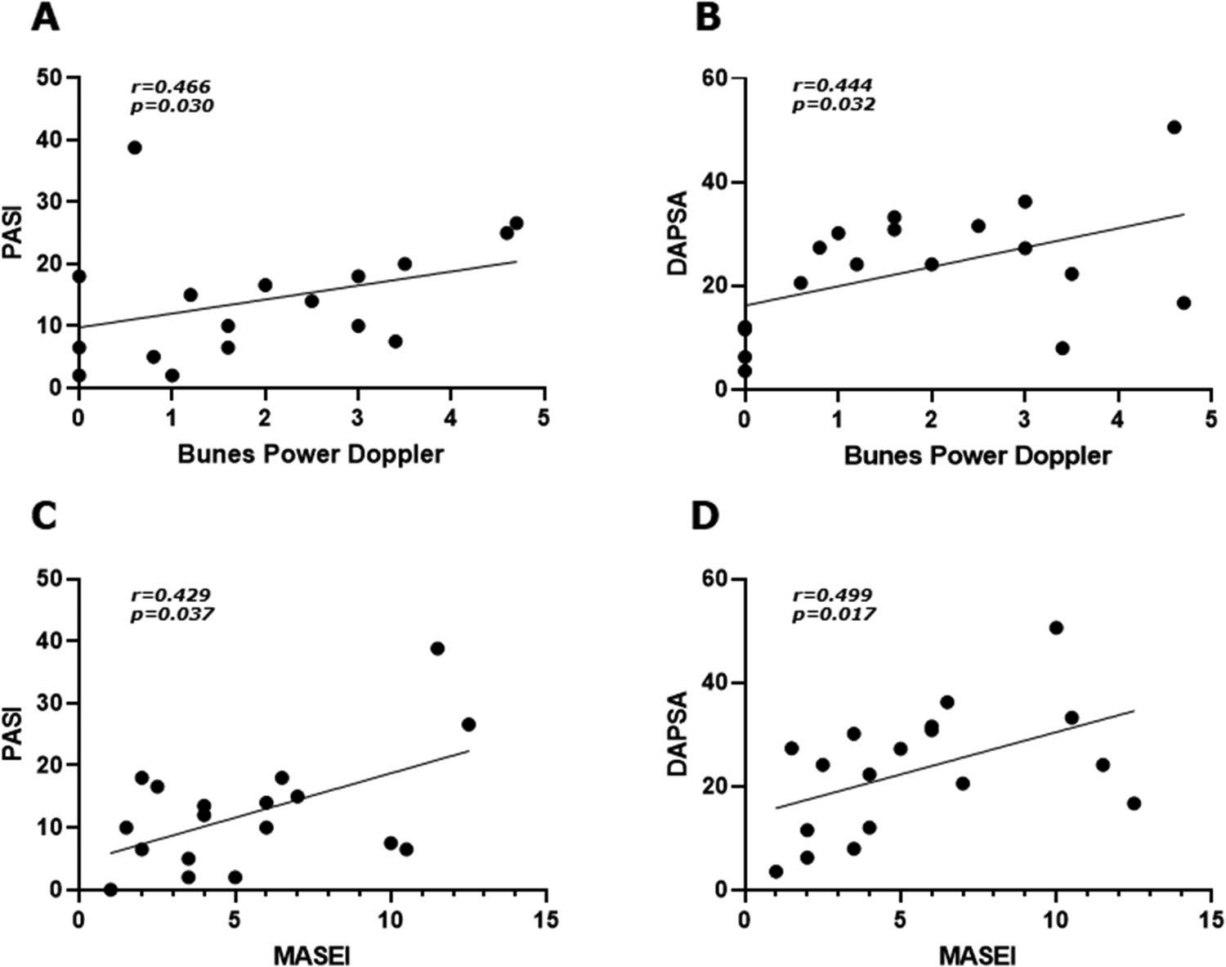
Correlations between HFUS findings at baseline and disease activity scores. In this figure, correlations analyses are reported; the values of BUNES PD significantly correlated with both PASI (*r* = 0.466, *p* = 0.030) and DAPSA (*r* = 0.444, *p* = 0.032), respectively, as shown in panels **A** and **B**. The values MASEI significantly correlated with both PASI (*r* = 0.429, *p* = 0.037) and DAPSA (*r* = 0.499, *p* = 0.017), respectively, as shown in panels **C** and **D**

The assessment of psoriatic nail involvement was performed before and after 24 weeks the administration of bDMARDs. The HFUS evaluation identified morphological and vascular nail findings in assessed patients at baseline. An increased blood flow was identified by PD, which was observed as elongated, dilated, and tortuous blood vessels to be attributed to an active inflammatory process. Assessing the structural changes, thickened matrix, inhomogeneous echogenicity nail bed, enlarged nail enthesis, loss of ventral and dorsal plate definition were also reported. After 24 weeks of the treatment, a reduction of the nail PD was observed, whereas the stabilization of the structural changes. Representative images of these findings are reported in Fig. 1. The nail HFUS abnormalities were also scored by BUNES before and after the treatment. Despite the trend, no significant reduction of the values of BUNES morphometry was observed before and after the treatment considering the whole cohort of treated patients with bDMARDs [baseline 0.62 (0.81) vs 24 weeks 0.28 (0.45), *p* = 0.578]. Assessing the findings retrieved by the HFUS assessment and considering the data for each drug, a trend for the reduction of these values was observed in patients treated with either IL-17 or IL-23 inhibitors. Specifically, no significant results were observed for the patients treated with IL-17 inhibitors [baseline 0.80 (0.79) vs 24 weeks 0.28 (0.66), *p* = 0.405], and for those treated with IL-23 inhibitors [baseline 0.80 (0.79) vs 24 weeks 0.28 (0.66), *p* = 0.424], as shown in Fig. 2 (panels A, B, and C, respectively). A significant reduction of the value of BUNES PD was reported before and after the treatment considering the whole cohort of treated patients with bDMARDs [baseline 1.92 (0.90) vs 24 weeks 0.88 (0.56), *p* = 0.044]. Despite a trend, no significant results were observed assessing the patients treated with IL-17 inhibitors [baseline 2.00 (1.21) vs 24 weeks 1.23 (0.98), *p* = 0.315], and for those treated with IL-23 inhibitors [baseline 1.92 (0.95) vs 24 weeks 0.98 (1.23), *p* = 0.119], as shown in Fig. 2 (panels D, E, and F, respectively). In addition, we estimated the association between the HFUS findings on nails with disease activity analysing both skin and joint involvement. The baseline values of BUNES PD correlated with PASI (*r* = 0.466, *p* = 0.030) and with DAPSA (*r* = 0.444, *p* = 0.032), respectively. These data are reported in Fig. 3 (panels A and B). The baseline values of BUNES morphometry did not correlate with these scores of disease activity.

### The effectiveness of bDMARDs in psoriatic enthesis involvement by HFUS

**Fig. 4:**
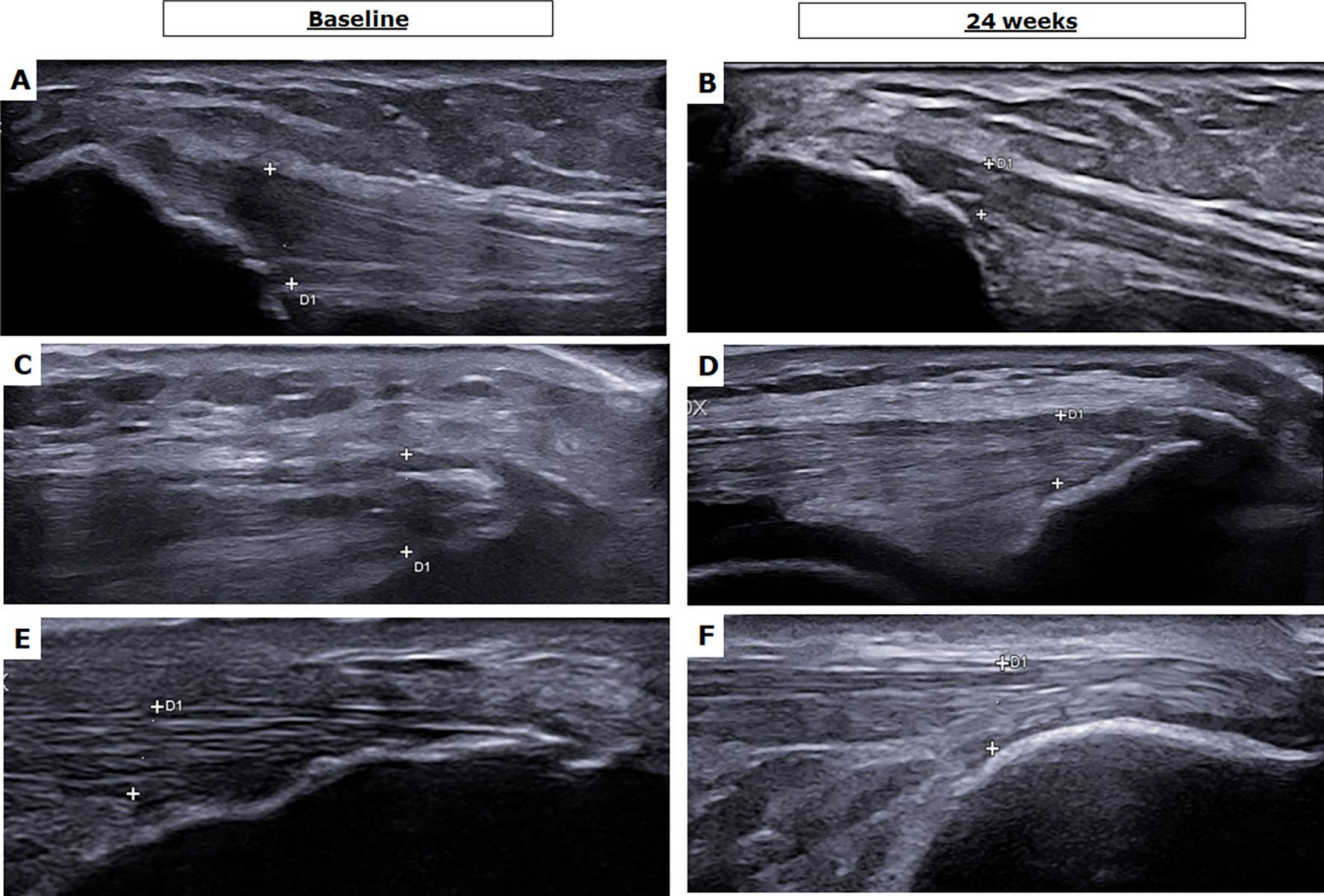
Representative images of entheses assessed by HFUS before and after 24 weeks of treatment. In this figure, an improvement of PsA enthesitis may be observed after the treatment in terms of tendon structure and thickness in representative evaluated patients as shown in panels **A** and **B** (enthesitis of the triceps, patient treated with IL-23 inhibitor), in panels **C** and **D** (enthesitis of the quadriceps, patient treated with IL-23 inhibitor), and in panels **E** and **F** (enthesitis of the Achilles tendon, patient treated with IL-17 inhibitor)

The assessment of psoriatic enthesis involvement was performed before and after 24 weeks the administration of bDMARDs. At baseline, the HFUS evaluation of entheses showed some modifications of the structure of the entheses as a hypoechoic aspect and thickness of the tendon, focal cortical erosion, and enthesis ossification. No significant difference was observed assessing the two sides of each evaluated tendon. However, an increase of the vascularity was infrequently reported by PD assessment of these sites. In addition, some irregularities of enthesis cortical bone profile, and enthesophytes were also described in these patients at baseline. After the 24 weeks of the treatment, an improvement was observed about the hypoechoic aspect and the associated thickness of the tendon. A stabilization of the structural changes was pointed out. Representative images of these findings are reported in Fig. 4. The enthesis abnormalities retrieved by the HFUS assessment were scored by MASEI before and after the treatment. A significant reduction of the value of MASEI was pointed out before and after the treatment considering the whole cohort of treated patients with bDMARDs [baseline 5.34 (3.56) vs 24 weeks 2.67 (1.87), *p* = 0.045]. Assessing the findings of the HFUS evaluation and considering the data for each drug, a trend for the reduction of the values for MASEI was observed in patients treated with either IL-17 or IL-23 inhibitors. Specifically, no significant results were observed for the patients treated with IL-17 inhibitors [baseline 6.00 (4.67) vs 24 weeks 2.22 (2.6), *p* = 0.083], and for the patients treated with IL-23 inhibitors [baseline 4.02 (2.45) vs 24 weeks 2.22 (1.98), *p* = 0.173], as shown in Fig. 2 (panels D, E, and F, respectively). In addition, we estimated the association between the HFUS findings on entheses with disease activity analysing both skin and joint involvement. The baseline values of MASEI correlated with both PASI (*r* = 0.429, *p* = 0.037) and DAPSA (*r* = 0.499, *p* = 0.017), respectively. These data are reported in Fig. 3 (panels C and D).

## Discussion

In this prospective proof-of-concept study, we assessed the effectiveness of IL-17 and IL-23 inhibitors in psoriatic nail and enthesis involvement evaluated by HFUS after 24 weeks of treatment in early naïve patients with PsA. To the best of our knowledge, this would be the first observation about the effectiveness of bDMARDs evaluated by HFUS in PsA in providing some novel insights in this context. Thus, an additional accurate imaging technique to assess these domains of PsA may be suggested in supporting the clinical evaluation to further improve the management of these patients. In fact, the ultrasonography is increasingly used in daily clinical practice due to its bedside utilization, ability to assess simultaneously multiple sites, and reasonable price [13]. Furthermore, several studies showed the higher value of ultrasound assessment over the physical examination in both nails and entheses [21–23]. The ultrasonography is useful in the assessment of the internal structures of the nails which could not be easily evaluated via clinical examination in spite of the magnification provided by a dermoscopic evaluation [23]. Similarly, although the enthesitis is easily assessed by clinical examination, the ultrasonography showed a better sensitivity and specificity in this evaluation [24]. On these bases, the European recommendations for imaging in spondylarthritis emphasized the use of musculoskeletal ultrasonography for the monitoring of peripheral involvement [25]. Thus, considering the technical advantages of HFUS in respect to commonly used frequency probes, a more sophisticated tool may be suggested in PsA to further improve the management of patients affected by these clinical manifestations. In addition, our reproducibility analysis showed excellent intra- and inter-observer reliability, confirming that our method yielded highly reproducible results. Despite the high ICC values (> 0.95 for both intra- and inter-observer comparisons), the test indicated a small but significant systematic difference between the observers’ measurements. This systematic variation may reflect subtle differences in measurement techniques or interpretations and underscores the need for careful standardization of measurement protocols in future studies. Nevertheless, this finding could not substantially compromise the overall reliability and validity of our results.

In this study, an improvement of some HFUS parameters was observed following the administration of bDMARDs in patients with PsA. In particular, we observed a significant decrease of the values of both BUNES PD and MASEI in these patients with both IL-17 and IL-23 inhibitors. In fact, a reduction of nail PD was observed following the administration of such drugs. Moreover, the improvement of tendons in terms of echotexture and margins was observed after the treatment. These data may further reinforce the findings about the efficacy of bDMARDs in PsA which are reported in randomized controlled trials as well as in real-life experiences [26–31]. Furthermore, HFUS parameters paralleled with the clinical response suggesting additional possible surrogates of the disease activity in patients with PsA. In fact, the values of both BUNES PD and MASEI correlated with PASI and DAPSA, which clinically reflect the disease extension on skin and joint, respectively. These data may further support the idea of the psoriatic disease continuum in simultaneously involving the skin and the joints according to shared pathogenic mechanisms [1, 2, 7, 8]. Conversely, in our cohort, the values of BUNES morphometry improved less, possibly suggesting a more useful score to assess the severity of nail involvement which could more reflect the damage of the psoriatic nail. In addition, a longer follow-up could be needed to fully evaluate the efficacy of bDMARDs on nail morphometry advocating further studies to entirely clarify this issue. Furthermore, our results may reinforce the importance about the assessment of psoriatic nail and enthesis involvement by HFUS in informing the physicians about the effectiveness of bDMARDs on these specific disease domains, which are difficult to be fully assessed by the clinical assessment in the heterogeneous context of PsA. Moreover, our preliminary findings could provide the basis for further studies, also, possible multicentre randomized controlled clinical trials allowing to compare HFUS findings along with disease activity, quality of life measures, and functional improvement following the administration of bDMARDs.

Despite providing some insights of HFUS in patients with PsA in assessing nails and entheses, some limitations should be acknowledged. In fact, the single-centre design and the relatively small sample size, even if we selected a very homogenous population of early naïve patients with PsA, may limit the generalization of our results and the external validity. In fact, we mainly focused on the feasibility of HFUS in evaluating the effectiveness of bDMARDs rather to derive definitive conclusions; on these bases a specific sample size has not been estimated. In addition, the lack of a control group could limit the ability to draw firm conclusions about the effectiveness of the bDMARDs compared with placebo. Furthermore, considering our specific study design, a possible observer bias could also occur in HFUS scoring limiting the generalization of the obtained results. Taking together these observations, the hypothesis generating nature of our prospective proof-of-concept study should be recognized. In addition, our findings about the assessment of psoriatic nails and enthesis involvement by HFUS could contribute to the advances of applying and implementing these new technologies in daily clinical practice [32–34], even if independent clinical validations should be still performed in furtherly corroborating the results of our study. Therefore, subsequent confirmatory studies are warranted to entirely elucidate these issues based on our preliminary findings to improve the management of these patients towards a more personalized treatment [35, 36]. In addition, a close collaboration between clinicians and radiologists should be advocated to further improve the arrangement of future specifically designed and adequately powered studies in entirely clarifying these issues, also considering the assessment of patients with PsO at higher risk of developing PsA [6, 37–39]. Finally, we employed HFUS by using 27 MHz probes to assess nails and entheses in patients with PsA. However, in this context, the role of ultra-high-frequency ultrasound (UHFUS) transducers could be further proposed as new development. In fact, UHFUS transducers (from 30 to 100 MHz) may provide higher resolution in facilitating the visualization of superficial anatomical layers [40–42]. Therefore, the high spatial resolution and the realizable excellent image quality may allow a wider use of these novel high-frequency ultrasound techniques, which may have the potentiality to bring further innovation in the imaging of psoriatic nail and enthesis involvement.

In conclusion, in this prospective proof-of-concept study, the assessment by HFUS may provide an additional more accurate information about the effectiveness of bDMARDs in nail and enthesis involvement in patients with PsA. Our study may also contribute to the growing evidence about the effectiveness of both IL-17 and IL-23 in the context of PsA by the evaluation of specific disease domains, which could be not fully evaluated by the clinical examination. Furthermore, the ultrasound may be considered a relevant part of the clinical practice as a feasible and effective imaging technique allowing a more accurate disease definition and monitoring of treatment response. Further experiences are needed to fully evaluate the role of HFUS in PsA by specifically designed and adequately powered studies, with the possibility of use in the context of multicentre randomized controlled clinical trials, allowing to compare HFUS findings along with disease activity, quality of life measures, and functional improvement following the administration of bDMARDs, to independently corroborate our preliminary findings.

## Data Availability

All data relevant to the study are included in the body of the article.
